# Did primary spontaneous pneumomediastinum risk factor alter in the period of COVID-19 pandemia?

**DOI:** 10.1093/icvts/ivab312

**Published:** 2021-11-25

**Authors:** Cemal Aker, Celal Buğra Sezen, Ayşegül İnci Sezen, Mustafa Vedat Doğru, Merve Özbek, Muzaffer Metin, Levent Cansever, Mehmet Ali Bedirhan

**Keywords:** Pneumomediastinum, Coronavirus disease 2019

## Abstract

**OBJECTIVES:**

In this study, we aimed to establish risk factors for primary spontaneous pneumomediastinum associated with Coronavirus disease 2019 (COVID-19) and reveal those which are significant.

**METHODS:**

The study included 62 patients with primary spontaneous pneumomediastinum who presented to our hospital between 11 March 2020, the date of the first-reported COVID-19 case in our country, and 3 January 2021. Of these, 14 patients (22.6%) had COVID-19 and 48 patients (77.4%) did not have COVID-19.

**RESULTS:**

Of the 62 patients included in the study, 41 (66.1%) were male and 21 (33.9%) were female. The mean age was 28.90 ± 16.86 (range, 16–84) years. The most common symptom at admission was chest pain (54.8%). The mean age of the patients with COVID-19 was 39.35 ± 23.04 years and that of the patients without COVID-19 was 25.85 ± 13.45 years (*P* < 0.001). In receiver-operating characteristic curve analysis, the area under the curve for age was 0.785 (95% confidence interval: 0.648–0.922) and the optimal cut-off value was 24 years for COVID-19-positive patients. The highest sensitivity and specificity values were 0.857 and 0.729. Twelve (85.79%) of the COVID-19-positive primary spontaneous pneumomediastinum patients were aged 24 years or older (*P* < 0.001). Five patients (8.1%) had positive severe acute respiratory syndrome coronavirus 2 polymerase chain reaction test but no abnormal findings on computed tomography.

**CONCLUSIONS:**

Having an age of more than 24 years was associated with a higher prevalence of pneumomediastinum in COVID-19 patients and emerged as an important risk factor. Multicentre studies with more cases are needed to determine whether pneumomediastinum is associated with additional other risk factors related to COVID-19.

**Figure:**
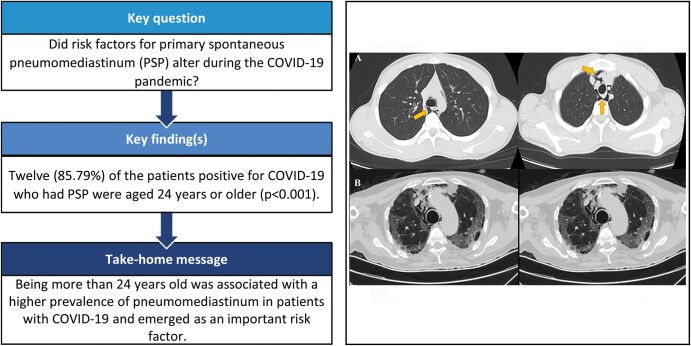

## INTRODUCTION

Coronavirus disease 2019 (COVID-19), caused by severe acute respiratory syndrome coronavirus 2 (SARS-CoV-2), was first detected in Turkey on 11 March 2020. After the World Health Organization (WHO) officially declared the pandemic, all medical conditions started to be treated according to algorithms appropriate for COVID-19 [1]. The medical community is continuously learning new information about COVID-19 and identifying uncommon clinical courses associated with COVID-19 is important to improve treatment and management of this new disease.

Pneumomediastinum, defined as the presence of free air in the mediastinum, is generally divided into 2 groups. Cases in which there is no apparent cause are classified as primary spontaneous pneumomediastinum (PSP), whereas secondary pneumomediastinum can be attributed to specific causes such as trauma, infection and oesophageal perforation. Alveolar damage often occurs in pneumomediastinum due to increased intrathoracic pressure caused by forceful coughing or Valsalva manoeuvre. The diagnosis of PSP is usually made in the absence of an obvious cause of air leak into the mediastinum (e.g. blunt trauma, oesophageal or tracheal perforation, mechanical ventilation, barotrauma) [2, 3].

Since the onset of the COVID-19 pandemic, there have been case reports and studies with limited numbers regarding the coexistence of PSP [4, 5]. However, these studies did not evaluate the relationship between PSP and COVID-19 and the risk factors associated with PSP. Therefore, there is no clear consensus in the literature on the relationship between COVID-19 and pneumomediastinum. In this study, we aimed to establish risk factors for PSP associated with COVID-19 and reveal those which are significant.

## ETHICAL STATEMENT

This study was approved by the institutional review board of Science of Health University, Yedikule Chest Diseases and Thoracic Surgery Education and Research Hospital (decision no: 2020–63) and conducted in accordance with the principles of the Declaration of Helsinki.

## MATERIALS AND METHODS

**Figure 1:**
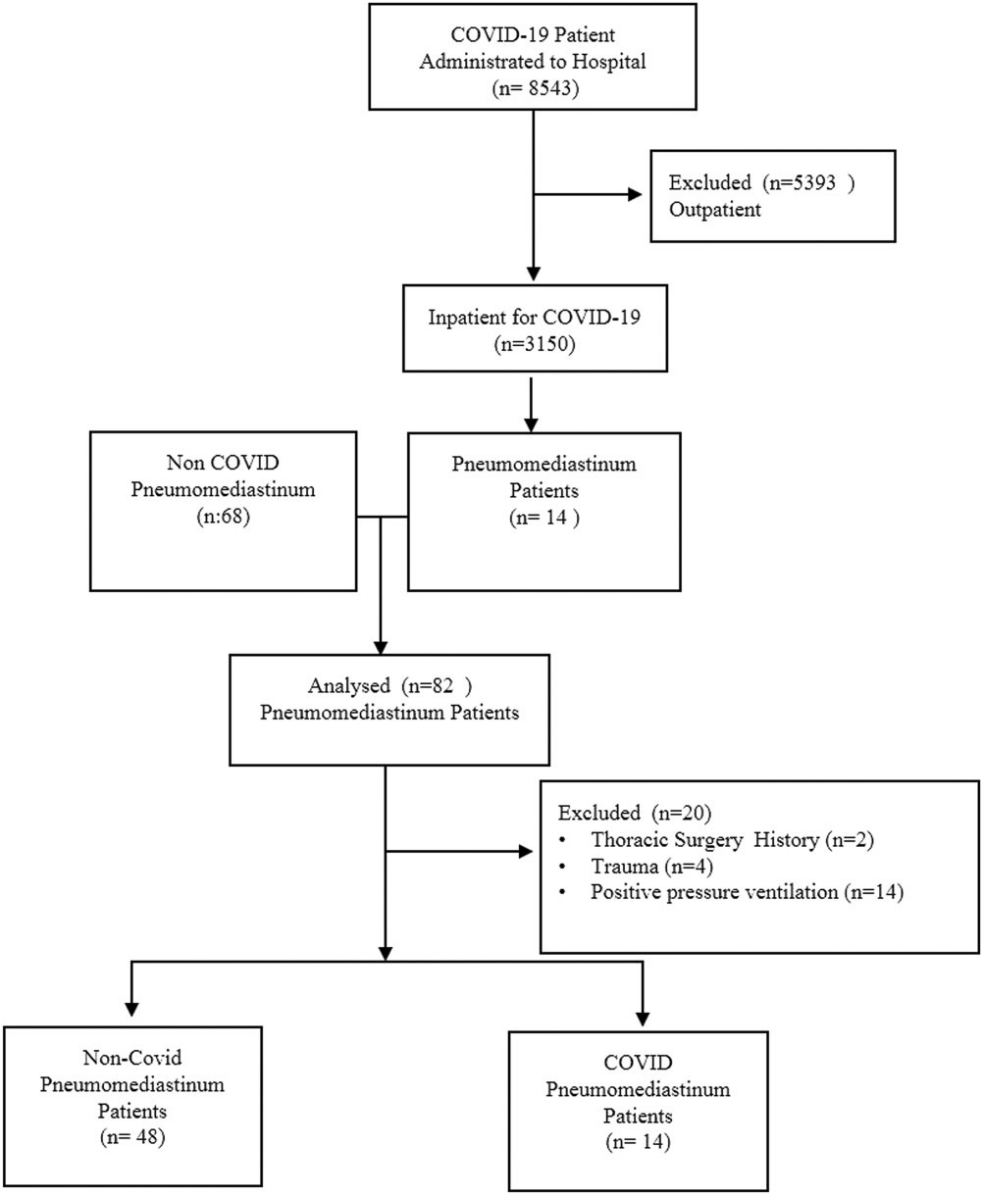
Study flow chart. COVID-19: coronavirus disease 2019.

**Figure 2:**
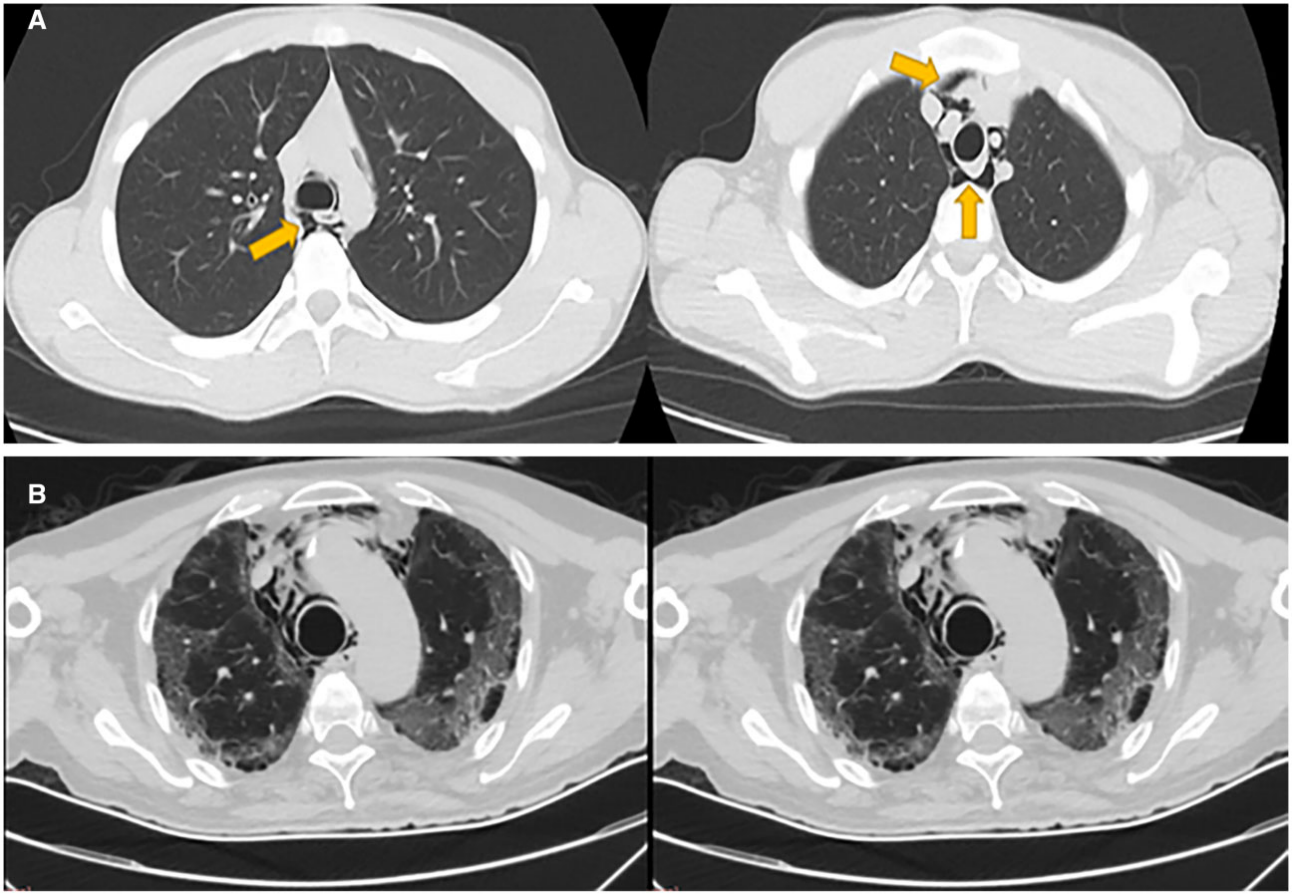
(**A**) Chest computed tomography image of a severe acute respiratory syndrome coronavirus 2 polymerase chain reaction positive patient with pneumomediastinum but no typical radiological findings of coronavirus disease 2019 (patient 2). (**B**) Chest computed tomography image of a severe acute respiratory syndrome coronavirus 2 polymerase chain reaction positive patient showing pneumomediastinum and diffuse ground-glass opacity associated with coronavirus disease 2019 (patient 3).

The study included patients with PSP who presented to our hospital between 11 March 2020, the date of the first-reported COVID-19 case in our country, and 3 January 2021. Between these dates, 8543 patients were diagnosed with COVID-19 and 3150 of these patients were admitted for inpatient treatment. A total of 82 patients were diagnosed with pneumomediastinum during this period. Exclusion criteria were history of thoracic surgery or trauma, risk factors for pneumomediastinum and being treated with positive pressure ventilation. Patients with risk factors for pneumomediastinum were excluded to avoid the heterogeneity in the study. As a result, 62 PSP patients were evaluated in the study (Fig. 1). The patients were separated into 2 groups: those who had radiological findings consistent with COVID-19 and/or positive SARS-CoV-2 polymerase chain reaction (PCR) were classified as COVID-19 positive, and patients who had negative SARS-CoV-2 PCR test and no signs of COVID-19 on chest computed tomography (CT) were considered COVID-19 negative. Chest CT findings specific for COVID-19 included ground-glass opacities with areas of consolidation and bilateral multifocal lesions located predominantly in the lower lobe (Fig. 2A and B). Non-COVID group patients (*n*: 48) were diagnosed as PSP in the study whereas, 14 patients in the other group had COVID-19 pneumomediastinum (Fig. 1).

### Patient selection

Prior to hospital admission, all patients underwent chest CT and SARS-CoV-2 PCR tests were ordered regardless of whether they reported symptoms of active COVID-19 infection.

Haemogram, biochemistry and coagulation tests were performed and ferritin, D-dimer and fibrinogen levels were analysed in all patients. Based on the evaluation of radiological and laboratory findings and PCR test results, 14 of the patients were diagnosed as having COVID-19 and treated accordingly. Charlson comorbidity index was used for the standardization of the patient comorbidities [6].

### Patient follow-up and treatment

In accordance with the COVID-19 treatment protocol published by the Ministry of Health on 26 March 2020, all patients regardless of clinical severity received hydroxychloroquine (400 mg twice a day as loading dose, followed by 200 mg twice a day for maintenance) and oseltamivir (75 mg twice a day for 5 days) as primary treatment. Azithromycin was not initiated routinely due to its interaction with hydroxychloroquine but was added in selected cases according to the physician’s decision. Favipiravir was recommended for patients who exhibited clinical progression despite primary treatment. However, due to limited drug stocks and supply issues, favipiravir therapy was not available for patients outside of intensive care units.

In the European Society of Intensive Care Medicine Surviving Sepsis Campaign’s initial guidelines for the management of severe COVID-19 published on 20 March 2020, corticosteroid therapy (methylprednisolone 1–2 mg/kg/day for 5–7 days) only for mechanically ventilated acute respiratory distress syndrome patients was weakly recommended based on low-quality evidence. Therefore, corticosteroid treatment was not initiated in any of the patients.

As a result of increasing experience with COVID-19 and advances in the literature, the treatment protocol was updated in July 2020, after which patients with asymptomatic infection and mild to moderate pneumonia were treated with hydroxychloroquine and azithromycin. Favipiravir was initiated in severe pneumonia. During this period, corticosteroid therapy was not included in the Ministry of Health protocols.

According to the COVID-19 treatment protocol issued in October 2020, favipiravir treatment was initiated in all patients and hydroxychloroquine and azithromycin were not used in any patients. Patients with symptoms consistent with secondary bacterial pneumonia and laboratory findings supporting bacterial pneumonia were treated with third-generation cephalosporin. The antibiotherapy spectrum was expanded if necessary based on follow-up. For patients who showed progression despite 5 days of favipiravir therapy, favipiravir was continued for a total of 10 days.

In the August 2020 protocol, treatment was revised to favipiravir therapy combined with corticosteroid 40 mg/day for patients requiring oxygen support (SpO_2_ <90%). Due to the immunosuppression effect in all patients receiving corticosteroids, third-generation cephalosporin was initiated.

Additionally, independent of treatment protocols and oxygen need, all patients received 4 l/min nasal oxygen for pneumomediastinum. None of the patients included in the study required additional surgical intervention during follow-up for the treatment of PSP. All patients were followed up with conservative treatment.

The data underlying this article will be shared on reasonable request to the corresponding author.

### Statistics

Descriptive statistics were used to summarize demographic and clinical data. Relationships between categorical data were evaluated using chi-square (χ^2^) test or, in case of values below 5 in any cells of table, Fisher’s exact test. Analysis criteria were pre-specified. Receiver-operating characteristic (ROC) curve analysis was performed to determine the optimal cut-off value for age. Results with *P*-value <0.05 were considered statistically significant. Analyses were performed using SPSS version 22 software (IBM Corp, Armonk, NY, USA).

## RESULTS

Of the 62 patients included in the study, 41 (66.1%) were male and 21 (33.9%) were female. The mean age was 28.90 ± 16.86 (range, 16–84) years. Fourteen patients (22.6%) in the study were COVID-19 positive, whereas 48 patients (77.4%) were COVID-19 negative. The mean length of hospital stay was 2.61 ± 2.75 (range, 1–10) days. Symptoms included chest pain in 34 patients (54.8%), dyspnoea in 29 patients (46.8%), cough in 19 patients (30.6%), headache in 9 patients (14.5%), fever in 8 patients (12.9%), myalgia in 7 patients (11.3%) and gastroenteritis in 2 patients (3.2%). The demographic characteristics of pneumomediastinum patients with and without COVID-19 are compared in Table 1.

**Table 1:** Comparison of demographic characteristics

Variable		COVID-19 negative, *n* (%)	COVID-19 positive, *n* (%)	*P*-value
Age (mean ± SD)		25.85 ± 13.45	39.35 ± 23.04	**0.001^a^**
Age (years)	<24	35 (72.9)	2 (14.3)	**<0.001^b^**
	>24	13 (27.1)	12 (85.7)	
Gender	Male	31 (64.6)	10 (71.4)	0.63^b^
	Women	17 (35.4)	4 (28.6)	
Smoking	No	22 (45.8)	3 (21.4)	0.10^b^
	Yes	26 (54.2)	11 (78.6)	
CCI	0	40 (83.3)	9 (64.3)	0.12^b^
	≥1	8 (16.7)	5 (35.7)	
Length of hospital stay (days), mean ± SD		1.23 ± 0.42	7.36 ± 1.90	**<0.001^a^**

CCI: Charlson comorbidity index; COVID-19: coronavirus disease 2019; SD: standard deviation. Bold values indicates statistically significance.

a Mann–Whitney *U*-test.

b Chi-square test.

**Figure 3:**
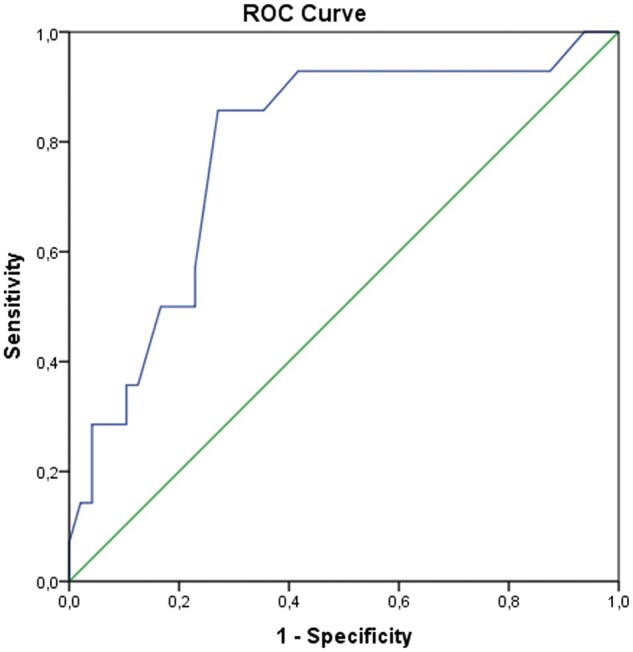
ROC curve analysis of age for the prediction of coronavirus disease 2019 positivity in pneumomediastinum patients. ROC: receiver-operating characteristic.

We performed ROC curve analysis because there are no standard cut-off values for COVID-19-positive PSP patients in the literature. In ROC curve analysis, the area under the curve for age was 0.785 (95% confidence interval: 0.648–0.922) and the optimal cut-off value was 24 years. The highest sensitivity and specificity values were 0.857 and 0.729. Figure 3 shows the ROC curve analysis.

The mean age of the patients with COVID-19 was 39.35 ± 23.04 years and that of the patients without COVID-19 was 25.85 ± 13.45 years (*P* < 0.001). There were 5 patients (8.1%) with positive SARS-CoV-2 PCR test and no abnormal findings on chest CT. The most common symptom was chest pain. The COVID-19 patient clinical findings at admission are shown in Table 2.

**Table 2:** The patient symptoms at presentation

	Age (years)	Sex	Dyspnoea	Chest Pain	Headache	Gastroenteritis	Cough	Myalgia	Fever
1	65	Female	−	+	+	+	−	+	+
2	18	Male	+	+	−	−	−	+	+
3	67	Male	+	+	+	−	+	+	+
4	78	Male	+	+	+	−	−	−	+
5	84	Male	−	+	+	−	+		+
6	32	Female	−	−	−	−	−	−	−
7	25	Female	+	+	−	+		+	+
8	25	Female	+	+	+	−	−	−	−
9	25	Male	−	+	+	−	−	−	−
10	30	Male	−	+	−	−	−	+	+
11	28	Male	+	+	+	−	+	−	−
12	26	Male	+	+	−	−	−	+	−
13	25	Male	−	−	−	−	−	−	−
14	23	Male	+	−	−	−	+	+	+

+ Symptom present, ^−^Symptom absent.

Symptoms of ‘chest pain, headache, gastroenteritis, fever and miyalgia’ were statistically significantly more in COVID positive group of PSP (*P* < 0.05). A detailed comparison of both groups on symptoms is presented in Table 3.

**Table 3:** Comparison of clinical presentations

Variable	COVID-19 negative, *n* (%)	COVID-19 positive, *n* (%)	*P*-value
Dyspnoea	21 (43.8)	8 (57.1)	0.37^a^
Chest pain	23 (47.9)	11 (78.6)	**0.04** ^a^
Headache	2 (4.2)	7 (50.0)	**<0.001^b^**
Gastroenteritis	0	2 (14.3)	**0.04^b^**
Cough	15 (31.2)	4 (21.1)	1**^b^**
Myalgia	0	7 (50.0)	**<0.001b**
Fever	0	8 (57.1)	**<0.001^b^**

CCI: Charlson comorbidity index; COVID-19: coronavirus disease 2019; SD: standard deviation. Bold values indicates statistically significance.

a Chi-square test.

b Fisher’s exact test.

None of the patients in the study died. One patient received non-invasive mechanical ventilation due to respiratory failure associated with COVID-19. This patient was included in the study because pneumomediastinum was present at the time of admission.

## DISCUSSION

PSP was first described through an examination finding by Louis Hamman in 1939. Crepitation heard with every heartbeat on auscultation is called Hamman’s sign [7]. Macklin first explained the pathogenesis of pneumomediastinum as air escaping from the pulmonary alveoli into the interstitium and dissecting the bronchovascular sheath to create artificial channels through which it reaches the lung root and then the mediastinum [8]. It is believed that increased intrathoracic pressure caused by strong cough or Valsalva manoeuvre causes the air leaking from damaged alveoli to separate the interstitial tissue around the bronchus and cause pneumomediastinum. It may be undetectable by standard X-ray imaging and be overlooked. Definitive diagnosis is established with chest CT. PSP can be triggered by various mechanisms, but no causative factor can be identified in a substantial number of patients [2]. Sahni *et al.* [9] reported in their 2013 study that smoking, respiratory pathologies such as asthma and interstitial lung disease, and previous respiratory tract infection were predisposing factors for PSP. Our results did not reveal any predisposition associated with the comorbidities and smoking history of the patients in our study.

The most common complaints in PSP are chest pain, shortness of breath and subcutaneous emphysema. Although it is rare according to the literature, the frequency of PSP may be expected to increase during an epidemic affecting the respiratory system. Consistent with the literature, the most common symptom in our study was chest pain, at a rate of 54.8% [2].

From December 2019 to the present, COVID-19 patients continue to exhibit different clinical presentations. PSP was evaluated as a clinical picture that may occur during the follow-up of COVID-19 patients presenting to the emergency department and admitted for treatment [4, 10–13]. Eperjesiova *et al.* [5] reported 7 spontaneous air leaks (2 with pneumothorax and 5 with pneumomediastinum) out of 976 COVID-19 cases. It is not possible to reach definitive conclusions about the pathogenesis of mediastinal emphysema in COVID-19 cases based on the available data. One study drew a parallel between COVID-19 and the earlier epidemic of severe acute respiratory syndrome in terms of pneumomediastinum development [14, 15]. In light of the pathological findings of COVID-19-associated acute respiratory distress syndrome, severe alveolar damage has been implicated as the possible cause of pneumomediastinum [16].

COVID-19 is a complex, emerging disease, and research is ongoing to fully understand the underlying pathophysiology. Diaz *et al.* [17] showed that pneumomediastinum may be a rare complication associated with COVID-19. However, as the mean age of the COVID-19 patients increased, the prevalence of pneumomediastinum also increased. Quincho-Lopez *et al.* [18] investigated the relationship between pneumomediastinum and COVID-19 and reported that male sex and comorbidity may be risk factors pneumomediastinum. In our study; other than age, remaining factors such as comorbidity scores, smoking, sex and respiratory tract infection were not found to be associated with the development of pneumomediastinum in COVID-19 patients.

### Limitations

As this study was single-centred retrospective, the incidence and risk factors of pneumomediastinum in new cases are unknown. Because COVID-19 is new to the world and our country, definitive information about its role in the pathogenesis of pneumomediastinum is not available at present. The small number of patients and young age distribution in the study are another factor limiting the acquisition of more exact data. Due to the small sample size, we were not able to perform multivariable analysis and propensity score matching in this study. We were unable to evaluate the increase in PSP incidence due to COVID-19.

## CONCLUSION

PSP patients with COVID-19 admitted to hospital with significantly more frequent symptomatic clinical presentation. Having an age of more than 24 years was associated with a higher prevalence of pneumomediastinum in COVID-19 patients and emerged as an important risk factor. Multicentre studies with more cases are needed to determine whether pneumomediastinum is associated with additional other risk factors related to COVID-19.

**Conflict of interest:** none declared.

## Author contributions

**Cemal Aker:** Conceptualization; Data curation; Formal analysis; Investigation; Methodology; Writing—original draft. **Celal Buğra Sezen:** Formal analysis; Methodology; Writing—review & editing. **Ayşegül İnci Sezen:** Methodology; Writing—review & editing. **Mustafa Vedat Doğru:** Data curation; Methodology; Writing—review & editing. **Merve Özbek:** Data curation; Methodology. **Muzaffer Metin:** Data curation; Methodology. **Levent Cansever:** (Data curation; Formal analysis; Methodology; Supervision; Writing—review & editing. **Mehmet Ali Bedirhan:** Supervision; Writing—review & editing.

## Reviewer information

Interactive CardioVascular and Thoracic Surgery thanks Arunkumar A. Arasappa, Kaushal K. Tiwari and the other anonymous reviewers for their contribution to the peer review process of this article.

## ABBREVIATIONS

COVID-19: Coronavirus disease 2019
CT: Computed tomography
PCR: Polymerase chain reaction
PSP: Primary spontaneous pneumomediastinum
ROC: Receiver-operating characteristic
SARS- CoV-2: Severe acute respiratory syndrome coronavirus 2
